# Assessment of maternal and newborn services in Uganda based on the Effective Coverage framework

**DOI:** 10.1093/eurpub/ckac130.247

**Published:** 2022-10-25

**Authors:** C Bertoncello, P Bordin, M Fonzo, G Dall’Oglio, P Lochoro, G Putoto, F Tognon

**Affiliations:** 1 Hygiene and Public Health Unit, DCTVSP, University, Padua, Italy; 2 Doctors with Africa CUAMM, Uganda, Uganda; 3 Doctors with Africa CUAMM, Padua, Italy

## Abstract

**Background:**

The implementation of a health service does not necessarily equate to a health gain. Effective Coverage (EC) aims to capture the potential benefits of a health intervention by adjusting the crude coverage for quality. The aim of this study was to assess the EC of Antenatal Care (ANC), Institutional deliveries and Postnatal Care (PNC) in Oyam district, Uganda, considering the input (drugs and equipment) and the process dimension (components of care provided).

**Methods:**

The study involved 19 Health Centers (HC), 12 type II, 6 type III and 1 type IV, having a catchment area of 15.603 expected deliveries per year. The analysis covered the period between April and September 2021. Data on crude coverage were retrieved from the District Health Information Software-2. Data used to assess quality domains were extracted from checklists compiled during Supportive Supervisions and were summarized by readiness and likelihood of quality care indices. The crude coverage of the interventions was adjusted to calculate the input-adjusted and the quality-adjusted coverage.

**Results:**

The readiness index was 0.81 for ANC, 0.82 for institutional delivery and 0.88 for PNC, while the likelihood of quality of care was 0.73, 0.88 and 0.89 respectively. In all three areas, the loss of coverage was mainly due to lack of materials and equipment; HCs II showed lower quality indexes than HCs III, particularly for ANC (P = 0.007). Compared to the target population, EC was 40% for ANC4 visits, 48% for institutional deliveries and 77% for PNC visits. The gap between crude and EC was higher for ANC4 (-30%) compared with the one for institutional deliveries (-18%) and PNC (-23%).

**Conclusions:**

EC is a useful indicator for monitoring maternal and neonatal services in low-resource countries, bringing gaps in crude coverage to the surface. Supportive Supervision provides an opportunity to assess EC at the facility level without additional resources and to support health authorities in setting priorities.

**Key messages:**

- The application of EC framework can be adapted both at district and facility level, either to a single service or healthcare pathways, and guide public health intervention.
- Integration of Supportive Supervision data in the EC is an innovative approach and permit to include the quality of care dimension in the routine data collection.

